# Utilization of Biomass to Ash: An Overview of the Potential Resources for Alternative Energy

**DOI:** 10.3390/ma14216482

**Published:** 2021-10-28

**Authors:** Natt Makul, Roman Fediuk, Mugahed Amran, Mohammed S. Al-Akwaa, Karol Pralat, Darya Nemova, Kirill Petropavlovskii, Tatiana Novichenkova, Victoria Petropavlovskaya, Mikhail Sulman

**Affiliations:** 1 Department of Civil Engineering Technology, Faculty of Industrial Technology, Phranakhon Rajabhat University, Bangkok 10220, Thailand; natt@pnru.ac.th; 2 Polytechnic Institute, Far Eastern Federal University, 690922 Vladivostok, Russia; 3 Department of Civil Engineering, College of Engineering, Prince Sattam Bin Abdulaziz University, Alkharj 11942, Saudi Arabia; mugahed_amran@hotmail.com; 4 Department of Civil Engineering, Faculty of Engineering and IT, Amran University, Amran 9677, Yemen; 5 Department of Civil Engineering, Faculty of Engineering, Sana’a University, Sana’a 9671, Yemen; msa-ak@hotmail.com; 6 Institute of Faculty of Civil Engineering, Faculty of Civil Engineering, Mechanics and Petrochemistry, Warsaw University of Technology, I. Łukasiewicza 17, 09-400 Płock, Poland; Karol.Pralat@pw.edu.pl; 7 Peter the Great St. Petersburg Polytechnic University, 195251 St. Petersburg, Russia; nemova_dv@spbstu.ru; 8 Moscow State University of Civil Engineering (MGSU), 129337 Moscow, Russia; kspetropavlovsky@gmail.com; 9 Tver State Technical University, 170026 Tver, Russia; tanovi.69@mail.ru (T.N.); victoriapetrop@gmail.com (V.P.); sulman@online.tver.ru (M.S.)

**Keywords:** biomass, recycling, energy, fuel, energy model

## Abstract

Climate change and the potential depletion of fossil fuels have increased international demand for alternative and renewable energy sources. In terms of the energy sector, for example, most of the South-East Asian countries (SACs) have a large number of biomass sources due to their vast forest resources and agriculture-based economies. Thus, the critical review was aimed at highlighting the overview of biomass energy in South-East Asia as a dynamically developing region, in order to obtain economic and environmental benefits from the existing sources of biomass in the world. The current review analyzed the sources of biomass, as well as their energy potential, use, and management, based on reports from different countries, published studies, and scientific articles. In SAC, the main sources of biomass were found to be coconut residues, oil palm residues, sugar cane residues, rice straw, rice husks, wood waste, and firewood. The combined annual biomass potentials in the forestry and agricultural sectors in South-East Asia were approximately over 500 million tons per year and more than 8 gigajoule of total energy potentials. The study identified the challenges and barriers to using biomass in these countries to achieve sustainable use of biomass sources and recommended sustainable approaches to using biomass energy by comparing traditional uses of biomass. Smart grid technologies have ways for solutions for better electric power production and efficient ways for distribution and transmission of electricity. Smart grids require less space and can be more easily installed when compared to traditional grids because of their versatilities. Upcoming challenges include technology optimization for the following uses of biomass energy: direct combustion of woody biomass; pyrolysis and gasification of biomass; anaerobic digestion of organic waste to produce biogas; landfill gas production direct incineration of organic waste. The barriers in this technology are emissions of carbon and nitrogen oxides, unpleasant odors, as well as the uncontrolled harvesting of biomass, which can harm nature.

## 1. Introduction

In the South-East Asian countries (SACs), booming economies have significantly increased consumption of energy. During 2005–2030, the consumption of energy was anticipated to increase 2.6 times [1]. In 2040, the demand of energy will possibly increase by virtually 66.67%, representing 10% of the increase in international demands [2,3]. In the meantime, the energy-associated ecological pollution caused mainly by emissions of greenhouse gases from the energy industry will increase regionally as well as internationally. The SACs, particularly Vietnam, Thailand, the Philippines, and Indonesia, should start swiftly investing in renewable electricity supply forms because of the climate change impacts [4,5]. To fit this issue, it is preferable to adopt the sustainable biomass energy technologies, but the barrier to adoption of sustainable biomass energy technologies is in justifying the prepositions of value by the providers of the services, as well as the customers, followed by technology standards and regulatory constraints that obstruct the technologies of sustainable biomass energy. The planners and operators of the power system still face various challenges in the integration of sources of renewable energy into grids of the power system. Fossil fuels are vital to the economy of the SACs [6]. 

Factors such as a growing understanding of anthropogenic international causes of environmental degradation, eco risks, and volatility in oil prices motivate broad interests in locally generated alternative fuels, such as sustainable biomass energy. The demand for sustainable biomass energy in the SACs was increased by approximately 21% between 2019 and 2020 [6]. In the current political and economic climate in the SACs, the alternative fuel goals are to produce more energy in the sustainable biomass energy and minimize emissions of greenhouse gases. The phrase “life cycle analysis” is commonly applied in describing the summations of benefits and costs to balance greenhouse gases and energy efficiencies accrued in the generation and consumption of sustainable biomass energy. The sustainable biomass energy life cycle for this analysis purpose starts with the biomass plant and ends with biomass energy production. 

The energy demands for SACs are estimated to grow significantly between 2015 and 2050 [7]. That is a reflection of increased populations with restricted mediations to minimize the general building’s need. The new houses shall have electric appliances, like building-level heat pumps. A huge number of existing gas boilers shall have to be substituted by the heat pumps [8]. In addition, heating, ventilation, and air conditioning are critical to energy demand [9].

The growing requirements for electricity around the world is likely to require additional substations and grid upgrades, including biomass energy utilization. At the same time, it is necessary to take into account the dependence on the energy of the main power supply networks. Population growth, combined with an increase in the number of jobs as well as homes, is projected to lead to an increase in global energy demand [10]. 

**Figure 1:**
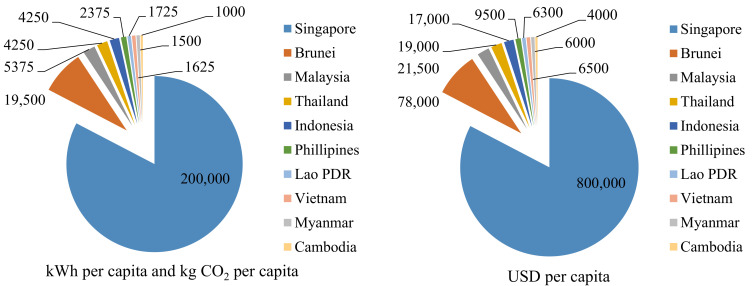
Relationships between per capita carbon dioxide emissions and buying power parities.

This review reveals a biomass energy plan that is a viable alternative to fuel. Low energy prices assumption [11], in this case, implies that structures have a high demand for electricity as investments in the retrofits of energy efficiencies that have been incentivized. The case leads to approximated reductions of sixty percent in emissions of carbon dioxide from levels in 1990. Because of the fast economic growth in SACs, the mean carbon emissions have gone up by more than 5% [11]. It is stated that growing buying energy parities result in a rise in consumption of per capita electricity, thus increasing the emissions of per capita CO_2_ [12]. Figure 1 indicates relationships among carbon dioxide emissions, per capita electricity consumption, and buying power parities [13,14,15,16,17,18,19]. CO_2_ is not a large source of greenhouse gas emission in South-East Asian nations in which agriculture and forestry are the main emission sources, even though CO_2_ is the biggest source of emissions of international greenhouse gases that originate significantly from the power industry [20].

For the appliances, there are approximately ten percent improvements in efficiencies and forty percent improvements. However, this is combined with more frequent use of electrical appliances due to the growing population. Life cycle analysis of individual biomass plants with state-particular biomass generation data notably yields different evaluations of greenhouse gases and energy than comprehensive analysis over significant geographic areas. The most fruitful and accessible ways in which biomass plants might decrease total emissions of greenhouse gases increase the energy efficiency of fuel.

This systematic review is mainly aimed at identifying possible studies of energy production from biomass carried out in the countries of South-East Asia. Based on the literature review, the trend in the use of biomass in the world was identified, including the problems and obstacles. The expected high population growth in South-East Asia is another reason why SACs need to study how to achieve net zero carbon.

## 2. Place of Biomass in the Energy System

**Figure 2:**
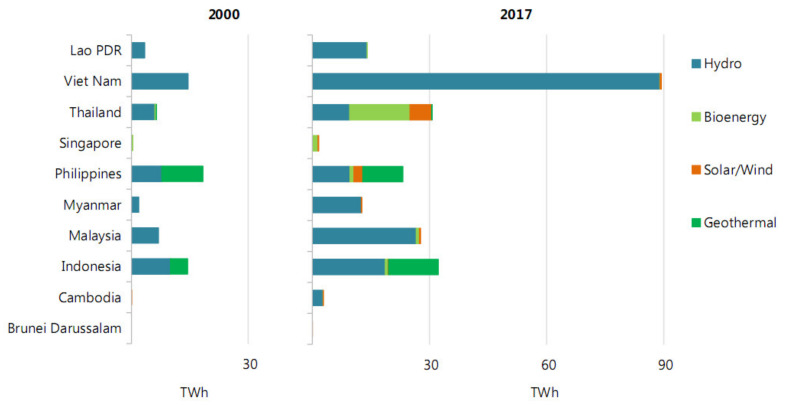
The renewable energy sources in SACs (2000–2017 years).

Figure 2 shows that in the first two decades of the 21st century, the consumption of renewable energy sources increased significantly. This can be traced especially clearly in the countries of South-East Asia for hydro resources, as the most developed type of energy in the world. At the same time, the use of renewable energy sources, such as wind and sun, began. Against this background, in Thailand, the use of biomass has increased tenfold, and, in many other countries, it has appeared as an alternative source of energy.

The detailed use of various sources of biomass in the countries of South-East Asia is listed in Table 1.

Table 1 shows that Malaysia is characterized by the use of oil palm waste; Vietnam—peanut and coconut shell; Cambodia and Laos—cassava stalk; Brunei, Myanmar and Indonesia—rice husk; Philippines—bagasse.

The heating value is one of the most important properties of biomass fuels for design calculations or numerical simulations of thermal conversion systems for biomass. There are a number of formulae proposed in the literature to estimate the higher heating value (HHV) of biomass fuels from the basic analysis data, i.e., proximate, ultimate, and chemical analysis composition [41,42]. In the papers [41,43], these correlations were evaluated statistically based on a larger database of biomass samples collected from the open literature.

## 3. Methodological Approach: Review Strategy

During recent years, since the beginning of the 21st century, the nation-particular data associated with bioenergy sources, power potentials, and usage in some nations, including Lao PDR, Myanmar, and Cambodia, have not been adequately accessible because the majority of SACs are emerging economies. However, the information was gathered to a viable level from reviewed papers, peer-reviewed research papers, reports from non-governmental organizations, and policy reports by institutions such as the Global Renewable Energy Agency, Global Energy Agency, UN Food and Agriculture Organization, World Bank and Asian Development Bank. The research then assessed and synthesized the biomass sources and the potential, usage, and management of biomass energy in the region to emphasize the possible usage of the available biomass sources in the SACs. In addition, the biomass usage challenges and barriers in the region were also explained [44]. Ultimately, the research recommended sustainable biomass energy approaches by comparing conventional biomass usage ways [40].

Many evidence-based interventions (EBIs) have been designed to improve energy production or reduce emissions of greenhouse gases. When implemented in diverse community settings, many EBIs nevertheless have portrayed restricted adoptions, reach, and sustainability [45]. These limitations are particularly noticeable in low-resource nations that serve energy disparity populations. Practitioners usually determine challenges with existing EBIs initially designed and tried with populations diverse from their target populations, and launch required adaptation to make the interventions more appropriate [46]. Most local adaptations to enhance fit for energy disparity populations are not well evaluated or documented, even though some EBIs have been extensively adapted for different populations and assessed [47]. As a result, empirical information is always lacking concerning the possible success of particular adaptation practitioners to be considered [48]. The researcher advocates for expansions in the emphases of adaptation study, from investigator-led interventions to studies that inform practitioners-lead adaptation. The researcher aims to inform study that facilitates effective adaptations and equitable implementations and deliveries of EBIs that improve energy security in SACs by presenting a study vision and strategies required to build this scientific area of biomass energy [40].

The systematic literature review revealed that evidence-oriented, biomass frameworks and innovative educational interventions for the promotion of biomass energy should assist policymakers in ensuring energy security and safety in SACs [49]. It is indicated that concentrated teaching approaches could have an impact on enhancing biomass production to integrate modern energy production methods. The research used cluster analytical techniques [50]. The multilevel data gathered are utilized to determine an evidence-based biomass energy framework to promote energy production and security in SACs. The sources are reliable because biasness is minimized by data triangulation. It indicated validity and reliability in the research study [40].

## 4. Demand for Electricity in SACs

The demand for electricity in SACs will grow by up to 15% between 2015 and 2050 [51]. With a growing population and, as a result, an increase in energy demand, it will be necessary to introduce new technologies, such as the utilization of biomass energy [52]. About a third of the heat demand of SACs shall be achieved by the use of heat networks and renewable energy like biomass production [53]. The installed systems of heat shall become steadily low carbons, and the sources of fuel shall show this through steadily renewable energy use and sources of domestic secondary heat like data centers and waste plants. This shall minimize the demands placed on the national gas and electricity networks.

**Figure 3:**
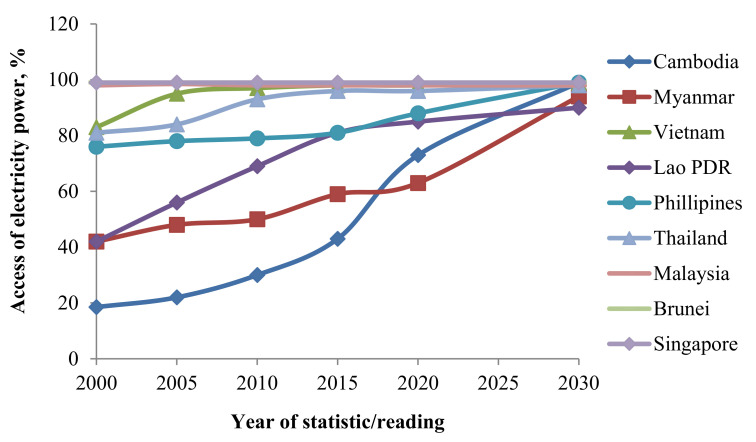
Access of electricity power in the SACs.

Sustainable biomass production will provide quality energy to fulfill the 21st century demands in SACs. It integrates technologies that consider the present energy challenges, such as direct combustion of woody biomass; pyrolysis and gasification of biomass; anaerobic digestion of organic waste to produce biogas; landfill gas production; direct incineration of organic waste [54,55]. The vital smart grid goal is to foster active decision making and participation of the customers as well as to develop functioning environments in which both the users of electricity and utilities influence one another. Users can influence the services in the smart grids through the addition of sources of distributed productions, like the solar panels (modules of photovoltaic-PV). Utilities can enhance reliabilities, effectiveness, and efficiency by the programs of demand response, adding distributed energy storage or productions at the substations and offering automated controls to the smart grids. For example, some of the South-East Asian states, like Thailand, Malaysia, Brunei Darussalam, and Singapore, have already gained access to 100% of power, as indicated in Figure 3 [51,53,56]. By 2030, other nations are meanwhile anticipated to achieve 100% of electricity access [51,57].

Representing 77.4% of the international supply of renewable energy and 10.40% of the globe’s total chief energy supplies, biomass power is by far the main source of renewable energy [5]. A major biomass feedstock supplier to markets like the U.S and Europe is Asia. However, new investments and opportunities in biomass are emerging within the region, especially in SACs [6]. With their plentiful biomass resources, SACs hold a strategic place in the atlas of international biomass energy [7]. In addition, the region is one of the biggest producers of wood and agricultural products. The quantities of biomass residues, according to conservative estimates produced from palm oil, sugar, and rice mills, are over 200–230 million tons per annum corresponding to coproduction potentials of 15–20 gigawatts (GW) [7].

SACs nowadays are quickly becoming striking markets for generating biomass as a source of energy [5]. Equal to 87% of the supply of renewable energies, biomass energies might offer 26% of supply of aggregate primary energy [6]. Most South-East Asian states are among the chief manufacturers of agricultural products like rubber, coconut, palm oil, canes, sugar, and rice; the most hopeful residues are wood residues, oil palm residues, sugarcane bagasse, and rice husks [5].

**Figure 4:**
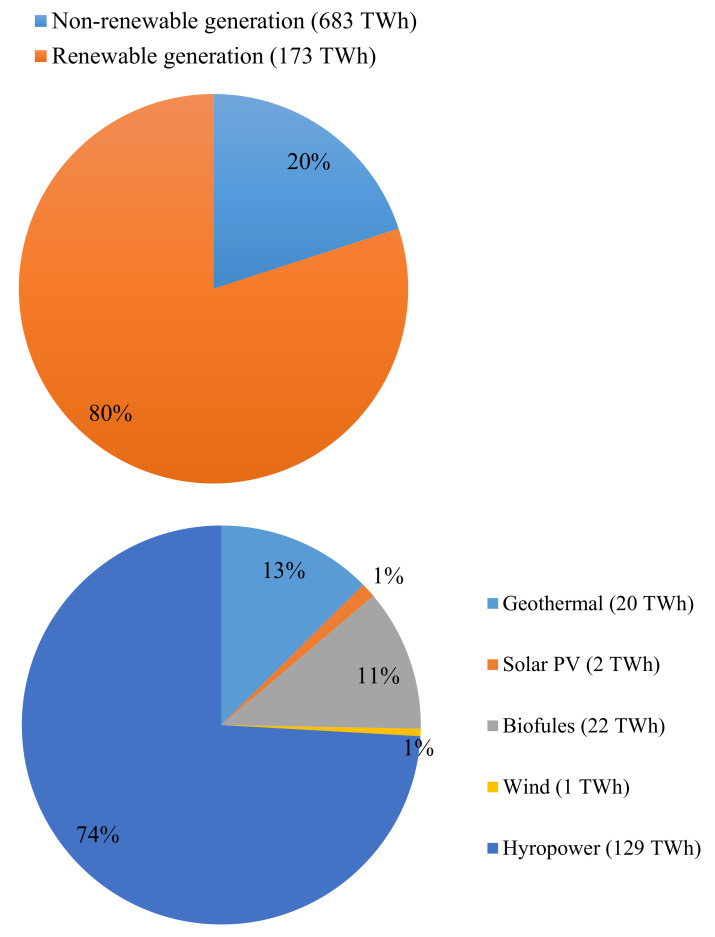
Share of renewable energy to total electricity generation (in 2014) in SACs.

The renewable energy share to the production of total electricity is indicated in Figure 4. The production of the aggregate power from non-renewable and renewable production totaled to about 856.0 Terawatt-hours (TWhrs) [57]. Out of these, 20% originated from renewable energies: accounting for wind (0.6%) [58], solar photovoltaic (PV) (1.2%) [59], geothermal (11.5%) [60], biofuels (12.6%) [61], and hydropower (74.1%) [53].

**Figure 5:**
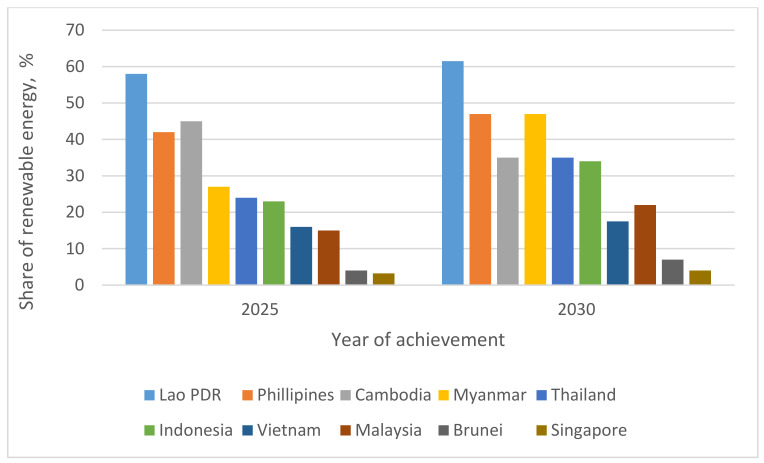
Potential portion of renewable energy in the entire main energy source in the SACs.

In total primary energy supplies, the possible renewable energies share in the Southern Asian nations is shown in Figure 5. The share of renewable energies in aggregate supply of primary energy at a country level appears to improve greatly, particularly in the nations that fully-phased out conventional applications of biomass, such as Myanmar, Indonesia, Lao People’s Democratic Republic (Lao PDR), and Cambodia [62]. The possible biomass energy share in the supply of aggregate chief energy is likely to be more than half of the total supply of primary energy by 2025 in Lao PDR (Figure 5).

Various studies have concentrated on the emission factors, policy, energy utilization, renewable energy sources, bioenergy, and sources of renewable energies [2,8,9,15,16,17,18,19], but mainly in particular nations like Philippines, Brunei Darussalam, Lao PDR, Cambodia, Malaysia, and Indonesia [19,20,21,22,23,24,25]. Thus, the review underlined the sources of biomass, management, and potentials of bioenergy in SACs in ensuring the sources of biomass and their energy usage are applicable to non-governmental organizations, policymakers, and scholars looking out for biomass energy as a crucial part of the sector of future renewable energy in SACs.

## 5. Current Status of Biomass in SACs

**Figure 6:**
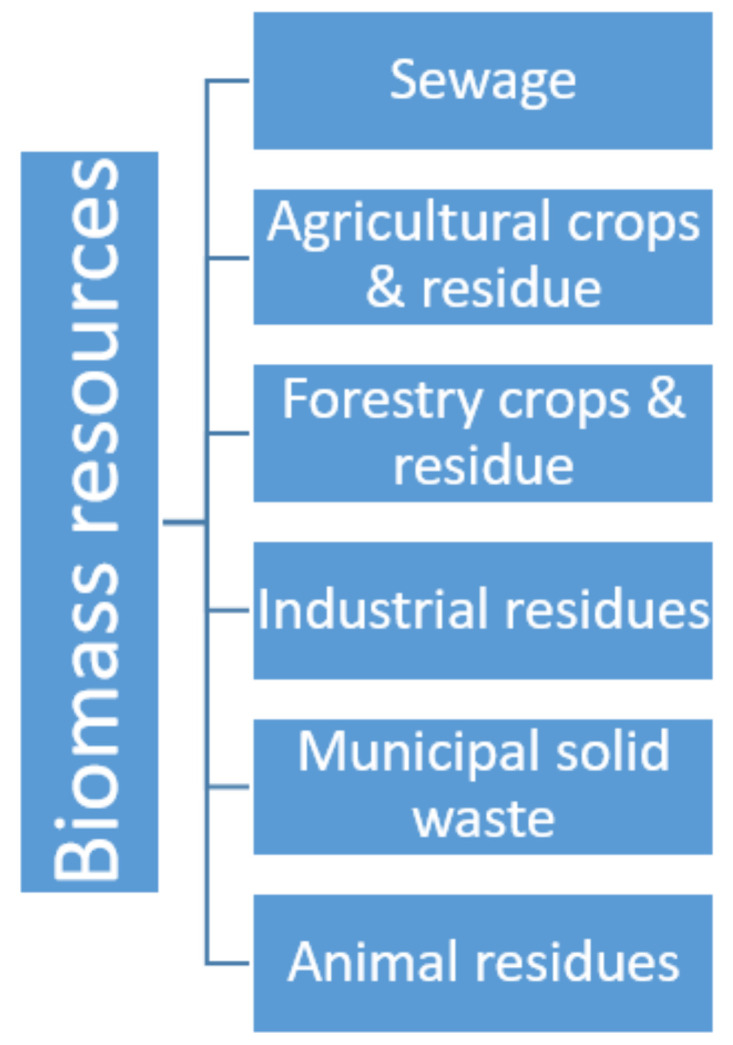
Manifold of biomass resources.

South-East Asian countries are located in tropical regions and have the capability of producing huge bioenergy amounts throughout the years. For the local people, bioenergy from forests makes up a specifically precious energy source in the domestic fuel form [16]. In addition, most of the region has plenty of agricultural wastes for sources of bioenergy because of their agricultural-oriented economies. For example, Myanmar has increased the bioenergy resources from the agricultural and forest sectors with an agriculture-oriented economy and 45% of forest covers. A careful assessment should be made of the potential for the use of the entire variety of energies of various biological resources, as illustrated in Figure 6. Sewage and solid waste is constantly generated in urban environments and can not only be a source of waste and pollution, but also an excellent and efficient raw energy material. The same can be said about agricultural waste, which is generated in huge quantities in tropical and equatorial regions. In SACs, the agriculture and forest resources are highlighted in Table 2.

In SACs, the bioenergy sources and their energy potentials are presented in Table 2. The biomass utilization in the SACs is described in Table 3. Per year, it is established that aggregate amounts of waste from the forest and agriculture sectors are approximated at more than 0.5 billion tons. In addition, the energy potentials of aggregate biomass in the nations have more than 8 billion gigajoules [63]. In each of the SACs, resources of agriculture and forest for biomass energy were briefly discussed in Table 2.

### 5.1. Cambodia

In Cambodia, 57% of the country’s total area (10.094 million hectares) is covered with forests. Thus, wood charcoal and woods are accountable for about 80% of aggregate energy consumptions. In rural areas, 94% of wood and charcoal are utilized for cooking, while 80% of the fuels are used in the urban areas. The other main bioenergy sources originate from agricultural wastes such as coconut fronts and shells, groundnut husks and shells, bagasse, cassava stalks, corns cobs, rice straws and husks [64]. The aggregate installed capacities are currently approximately 23.0 MW. By 2030, Cambodia has a plan of generating more than 75.0 MW of installed biomass capacities.

### 5.2. Myanmar

Covering 45% of the country with forests, Myanmar is an agricultural state [28]. Myanmar generates more than twenty million tons of paddies yearly. Thus, the nation’s main bioenergy sources are acquired mainly from the agriculture and forest sectors. The residents are largely dependent on solid bioenergy fuels because 70% of the people live in rural areas [63]. About 65.0% of the aggregate energy consumptions of Myanmar originate from bioenergy sources. From biogas and biomass, the aggregate capacity potentials are approximated at 4741 MW and 6899 MW, respectively [29]. The total installed capacities out of these have reached 115.0 MW [63].

### 5.3. Lao PDR

Lao PDR has plenty of biomass sources from the forest sectors, with over 65% of the country covered in forest [26]. About 80% of families depend on charcoal and firewood because of the people dwelling in rural areas. Charcoal and firewood have 68% of the chief energy supply in the rural areas. The agriculture sector might offer a wealth of bioenergy sources, in addition to the forest sector, because of its agricultural economy [30], as the installed bioenergy capacities is expected to reach about 40.0 MW by 2025. Also, Lao PDR is planning to accomplish 58 MW of biomass capacity [63].

### 5.4. Vietnam

Vietnam has plenty of bioenergy resources, being an agriculture nation with more than 300 GW theoretic capacity potentials [31]. Bioenergy is mainly used at household levels (76%) [63]. Only 24% of the biomass energies are utilized in combined heat and power (CHP) plants and small industrial boilers in sugarcane grinders [32]. The main bioenergy resources are coconut shells, coffee husks, peanut shells, cassava stems, maize residues, cane wastes, bagasse, rice straws and husks, and forest residues. In Vietnam, the major consumption potentials are aimed at industrial and municipal production energy factories. By 2020, Vietnam has achieved 500 MW combined capacity of biomass energy. By 2030, the country targets 2000 MW combined capacities [33].

### 5.5. Philippines

The Philippines government has looked at renewable energies for potential alternatives because the power needs of the country dominantly depend on fossil fuel imports [34]. Bioenergy, among the alternatives, is important to the Philippines as almost 30% of the energy for the one-hundred million citizens dwelling in the country originates from bioenergy and is largely utilized by the rural populations for household cooking [27,35]. In addition, with 276.70 MW of aggregate installed capacities around the Philippines, the bioenergy sector is quickly changing [35]. In the Philippines, applications of bioenergy account for virtually 15% of the chief energy applications [36].

## 6. Sustainable Energy Model

The integration of smart grid tools contributes to noteworthy enhancements in dependability. Grid operators can monitor power quality, which helps them in the early detection of unhealthy equipment. An additional advantage of an automated grid is its ability to self-heal, ensuring that it recovers quickly. It can start, stop, or reroute power flow to avoid further problems without manual intervention. The use of a Geographic Information System (GIS) helps in the network’s design, and this has been found to promote optimization of the grid infrastructure. GIS is a tool widely used in scheduling maintenance works and has been observed to save between 10 and 30 percent by reducing operational expenses [65]. Additional cost savings come from distributed generation by deferring improvement or establishing new transmission and distribution networks connected to the client loads. The system operator can integrate the massive volume of data on a real-time basis so that correct price indications may be produced and billing data can be gathered. The well-timed settlements of the vast number of new dealings can be achieved. The latter part can be facilitated with IoT and blockchain technologies [65].

A smart grid comes with security improvements that increase its robustness and resiliency for physical and cyber-attacks. This reduces the probabilities and concerns of human-made attacks (such as theft and infrastructure vandalism) and natural disasters. The Advanced Metering Infrastructure (AMI) fitted provides operators with real-time and two-way connection on personal customer statuses. This includes the ability to remotely connect and disconnect their loads while demand response components provide valuable information that is key in sustaining services when the delivery systems are stressed, decreasing the likelihood of outages. Grid operators are capacitated in deterring, detecting, mitigating, responding to, and restoring emergency occurrences. The use of intelligent components in the transmission infrastructure provides control capabilities of detecting attempts to attack the transmission systems and reduce the impact zone. A smart grid can also be fitted with video monitoring of transmission stations that shall prevent vandalism, sabotage, and theft activities [66].

All biomass should go through various phases to generate biomass energy [3]. All the phases require resources and infrastructures. The energy ratio produced by the resulting biomass energy combustions to power used in the process is known as the biomass fuel energy balance, or the net power gained. Various site-specific and independent variables determine the greenhouse gas and life cycle energy assessment of biomass energy fuels. When all aspects of biomass energy generation and applications of co-products are tightly integrated, particular agriculture aspects present opportunities for optimizing the energy efficiencies and greenhouse gas biomass fuel neutrality. When compared to gasoline, it is estimated that the current biomass energy production has about 1.6 net energy ratios and minimizes emissions of greenhouse gases by more than forty-five percent [1]. When all co-products are assumed to be fed to dairy cattle in wet forms within 64km of the distillation plants, the emissions of greenhouse gases decrease by 15%, and net energy ratios increase to 1.9 [67]. The impacts of substituting natural gases with biogas from the fermentation of corn are remarkable. Life-cycle analyses of biomass energy production are sensitive to inputs of nitrogen and lime [2]. Minimizing the nitrogen and lime inputs by applying and crediting possible basicity and nutrients into the output of corn-based ethanol further decrease emissions by 10% and increase the net energy ratios to 2.00 [2]. Biomass energy is renewable fuels generated from corn materials through the biomass process. Over 98% of the biomass energy typically contains E10 (90% gasoline and 10% ethanol) [67]. This oxygenates the fuel and minimizes pollutions [3].

Proper biomass energy management to protect its quality is essential to most energy sustainability definitions. While focusing on biomass energy resource sufficiency, in the development of sustainable energy, early definitions concerning the quality of energy define the hazardous energy capacity given the proper practices and plans of energy management. Early scholars explained the entwined energy quality and sustainable human development difficulties in that energy might be utilized to remove pollutions from a site. Yet, energy also delivers such hazards to users of downstream [67].

Sustainable quality of biomass energy includes the biomass energy and infrastructure sustainability utilized within the systems to make biomass energy quality sustainability a pressing matter for urban areas in SACs. The burdens to ensure high-level human energy quality are put heavily on the utilities of biomass energy. With the latest laws ratified in Asia reducing the energy disparity, the regulations and policy in SACs uphold the even management practices. Utilities of biomass energy should not be responsible for the treatment of excess contaminants caused by the lack of agricultural regulations. Management procedures and policy implementation should be considered in urban, ecosystem, agricultural, and industrial energy as one interlinked power system. Without balanced energy-quality management policies, the players accountable for energy quality degradation will have no incentives to reverse hazardous practices [68].

The smart grid of electricity and biomass renewable energy production in accommodating greater renewable power percentages would need traditional backup of large energy amounts and storage of ample biomass energy. For natural variation compensation, this would be necessary in the energy quantities generated depending on time of the day, seasons, and other elements, such as biomass amounts, at any given time. The adoption expenses for renewable resource sources are more expensive than they should be, since today’s electricity cannot deal with these variabilities. Renewable biomass energy allows a renewable energy resource for greater penetration accommodations with cost-efficient and effective procedures when improving energy quality and reliability. In recent years, the utilizations of renewable biomass energy resources in the smart grid systems have been increasing. Various significant plans and strategies have been executed in different parts of SACs, both in the developing and developed nations. Certain research studies indicate that these renewable biomass energy technologies can offer relatively low cost and reliable electricity services. The renewable biomass energy offers an alternative, to provide SACs with sustainable and clear energy that does not pollute the environment [69].

Blockchain in waste management is implemented in order to gain control over waste management. Blockchain technology is being implemented in many areas, like healthcare and entertainment, and has shown the capability on how it is improved and changed. Blockchain in waste management is a new and trending technology, which can be used for an error-free waste management and disposal. When the blockchain technology is implemented, certain areas like product manufacturing and the food industry can cut down the amount of waste produced. To achieve these goals in the best possible way, waste managers and policy makers use the “waste hierarchy”. From top (best option) to bottom (least favorable option) these options are: prevention, minimization, reuse, recycling, energy recovery (incineration), and storage (landfills) [70].

Energy storage is regarded as the major link in the transformation of the current infrastructure and operation of the electrical grid. Table 3 shows the comparison between capacity of biomass and biogas energies with the targeted project in several SACs. Academic sources have indicated that the regulatory and policy framework has limited the deployment and up-scaling of energy distributed storage systems into the electrical grid. Currently, regulatory structures are based purely on market systems with no proper pricing signals. This does not contribute to full deployment in the transmission and distribution services. Energy storage has capability in the role of power management, bridging, and stability, thereby acting as a ‘shock absorber’ in the electrical grid. Having energy storage not accorded full asset status recognition, such as generation, signals the economic argument on cost recovery. The key to energy storage is that it can provide revenue and increase economic benefits similar to generation if offered the status of generation, transmission, and distribution services. An example is where energy storage operators can dispatch to the highest-valued use, thus generating a higher return unlike just supplementing energy systems. In this sense, one can agree that a smart grid and net metering network, driven by energy storage, demand side management and interconnection will shape the future outlook of the utility market, creating an Internet of Energy [71].

**Table 3:** Comparison between capacity of biomass and biogas energies with the targeted project in several SACs.

Compartments	Capacity of Biogas Energy (MW)	Capacity of Biomass Energy (MW)			Refs
Country	Potential (MW)	Potential	Targeted	Installed	
Lao PDR	51 MW by 2025	>200	58 by 2025	≈40	[6,7,8]
Philippines	-	-	-	≈277	[24]
Myanmar	4741	6899	-	-	[27]
Vietnam	-	318,630	500 by 2020 2000 by 2030	270	[29,31]
Cambodia	-	≈19 GWh/year	73 by 2030	23	[33,36]
Singapore	-	-	-	220	[39]
Thailand	46	7000	3.630 GW by 2021	1610	[46,47]
Malaysia	-	29,000	-	211	[48]
Indonesia	278	50,000	-	312	[49]

The regulatory framework enforces the set price and has incentivized investment growth and increased deployment of energy storage technologies. Policy and regulation enable the value and potential from energy storage by allowing access to ancillary and other markets, thereby acting as a powerful tool to build a storage-based smart grid network [72].

The economic effect of the proposed technology for the use of biomass energy should be noted. This effect is clearly visible if we compare how much the commonly used technology of using alternative energy sources will cost with the technology proposed by the authors. This is important because the cost of implementing a technology is the basis for its application (Table 4).

Table 4 shows that despite the relatively high investment, construction time, and production and operating costs, there are a number of economic advantages of using biomass in the energy sector. Compared to other types of alternative energy, biomass cogeneration plants provide an operating life of up to 314 days a year, which is 2 to 5 times longer than for the use of wind and sun. The service life is up to 40 years, which is up to 2 times higher than that of other alternative energy sources.

Thus, the review noted the use of smart grid technology and GIS as a model for sustainable energy. But one should be aware that this is not an alternative to the conversion and use of biomass. Further optimizations of biomass conversion technology are needed, such as combustion, co-combustion, gasification, and pyrolysis to generate electricity [73,74].

## 7. Net Metering Using Biomass Energy

Net metering refers to power policies allowing electricity service clients to offset some or all of their electricity use with a self-produced one. This is achieved by utilizing a single meter to record the bi-directional flow of energy. The wide spread of grid-interactive distributed energy generation is being implemented and supported globally through the promulgation of different types of tariff policies and incentive programs, such as feed-in-tariffs and quota systems. Despite the decreasing cost of renewable energy, especially biomass energy, the transition had been prolonged mainly due to the absence of attractive policies and tax incentives for investors. The most probable sufficient policy incentive is known as net metering systems. Net metering systems accelerated installations of distributed energy generation by allowing the grid-connected consumer generation to offset their electricity consumption through grid feeds and compensate for any excesses. With net metering, customers are paid on surplus generation at a retail price. This encourages the extensive participation of consumers and prosumers. This is simply done through state utilities buying back surplus electricity from customers who are interconnected to the grid by net metering systems. In that sense, net metering is regarded as a favorable policy to help overcome the financial barriers faced by distributed generators. Globally, net metering has been implemented and in operation for the last two decades only because renewable energy resources are reaching grid parity. This is a sign that technologies are entering a competitive stage with conventional generation. The net metering policy promotes various least-cost technologies and brings cost structure down. Net metering is an integral part of policy formulation to adopt large scale renewable energy installations.

## 8. Commercialization of Biomass Technologies

Biomass can be commercialized for energy use in three main ways:

- Direct combustion in boilers (straw, firewood, pellets, wood chips);
- Co-combustion with traditional energy sources (fuel oil, coal, gas);
- Combustion of biomass processing products during fermentation or esterification (biogas, biodiesel, methanol, ethanol).

Energy resources of biomass can be divided into two groups: solid-phase energy carriers suitable for combustion; pyrolysis and steam-oxygen gasification into a mixture of carbon oxide and dioxide, hydrogen, and methane. This gas can be converted into electricity and heat using appropriate technologies. Biomass components are converted into liquid fuel and biogas, which is a mixture of 60% vol. methane and 40% CO_2_ [26].

Commercial biomass processing technologies:

1. Pyrolysis is the most common method of producing energy from biomass (90% of the world’s commercial energy production from biomass is due to the use of this technology), which is used for both heat and electricity. Combustion boiler plants are suitable for the processing of various types of biomass; mainly wood, wood chips, sawdust, and straw. The process is carried out at temperatures above 600 C and without access to air, the output of which is liquid biofuel. The best raw material for the pyrolysis process is wood, but, since this technology is only at the beginning of its development, it can be assumed that any type of biomass can be converted in the pyrolysis process [29].

2. Gasification is a thermochemical transformation process, which differs from combustion in that the product of the process is not heat, but gas, which, after combustion, provides the desired heat energy. The gas can also be used in special turbines to generate electricity. The advantage of gasification is the high commercial efficiency of the process, reaching 50% [75].

3. Cogeneration is the process of generating heat and electricity at the same time. In cogeneration systems, lower emissions of pollutants are achieved [76].

4. Biochemical processes—some forms of biomass containing large amounts of water are used in the fermentation process, where the decomposition product of the biomass is the alcohol used to make biofuels. Methane fermentation processes are also used, the product of which is biogas (a mixture of methane and carbon dioxide). For commercial energy purposes, the fermentation process uses animal manure, food waste, and household waste in landfills and sewage [30].

## 9. Applicability of the Review to the Whole World

Biomass utilization is a global problem. All over the world, millions of tons of waste of various vital activities are present and accumulate, in particular, in sewers, in agricultural areas, in forests. A particular danger is caused by the avalanche-like accumulation of urban waste, which has to be removed to the constantly expanding waste disposal sites. With unreasonable waste management, huge areas are taken out of circulation, which, moreover, turn out to be contaminated for many tens of years. Although this review was based only on the results of South-East Asia, it is easy to trace these trends and extend them to the rest of the world. The efficient use of biomass energy will help not only in an effective global waste management strategy, but will also make it possible to make a tangible contribution to the green energy of the population of planet Earth.

Upcoming challenges include technology optimization for the following uses of biomass energy: direct combustion of woody biomass; pyrolysis and gasification of biomass; anaerobic digestion of organic waste to produce biogas; landfill gas production direct incineration of organic waste. The barriers in this technology are emissions of carbon and nitrogen oxides, unpleasant odors, as well as the uncontrolled harvesting of biomass, which can harm nature.

## 10. Conclusions

The presented review attempted to investigate the possibility of using biomass energy on a global scale using the example of the countries of South-East Asia. Because of a comprehensive analysis of modern literature, the following main conclusions were made:

1. Utilization of biomass over the past two decades has made a huge leap forward. In particular, in Thailand, since 2000, the amount of energy produced from these sources has increased several dozen times, and in Laos, Vietnam, the Philippines, Myanmar and other countries, the use of biomass energy has started from scratch.

2. The review highlighted biomass sources, governance, and bioenergy potential in SACs to ensure that biomass sources and their energy use are applicable to non-governmental organizations, policymakers, and scientists who view biomass energy as a critical part of the renewable energy sector of the future in South-East Asia.

3. It is estimated that in the countries of South-East Asia, the total volume of waste from the forestry and agricultural sectors per year is approximately more than 0.5 billion tons. In addition, the energy potential of the total biomass in these countries exceeds 8 billion gigajoules.

4. The use of biomass is promising for use in smart energy systems, which will contribute to maximum energy savings.

5. Regulatory adjustments are needed to ensure price compliance and stimulate investment growth and wider adoption of energy storage technologies. Policy and regulation leverage the value and potential of energy storage by opening access to ancillary and other markets, thereby acting as a powerful tool for building a grid of storage-based smart grids.

6. Net metering refers to an electricity policy that allows customers providing electricity services to offset part or all of their electricity consumption from their own electricity. This is achieved by using a single meter to record bi-directional energy flow.

7. The use of biomass is a global problem. Although this review was based only on findings from South-East Asia, these trends are easy to trace and replicate to the rest of the world. The efficient use of biomass energy will not only help in an effective global waste management strategy, but will also make a tangible contribution to the green energy of the people of planet Earth.

8. Upcoming challenges include technology optimization for the following uses of biomass energy: direct combustion of woody biomass; pyrolysis and gasification of biomass; anaerobic digestion of organic waste to produce biogas; landfill gas production direct incineration of organic waste. The barriers in this technology are emissions of carbon and nitrogen oxides, unpleasant odors, as well as the uncontrolled harvesting of biomass, which can harm nature.

## Figures and Tables

**Table 1:** Resources of biomass and the potential production of energy.

Country	Year	Type of Source (Biomass)	Content of Energy (MJ/kg)	Yearly Production (Million Tons/Year)	Potential Production of Energy (Million GJ/Year)	Refs
**Malaysia**	2009	Oil palm bunch	21.520	38.550	239.170	[20,21,22]
		Sugarcane top and trashier	17.450	0.211	1.103	
		Bagasse	18.110	0.204	1.467	
		Oil palm fiber	22.070	1.320	97.420	
		Coconut shell	20.150	0.735	1.319	
			19.600	0.166	2.701	
		Oil palm shell	23.510	4.410	69.590	
		Coconut frond	19.600	0.103	1.655	
		Coconut empty bunches	19.600	0.022	0.347	
**Vietnam**	2010	Forest residues	-	11.000	-	[22,23,24,25,26]
		Rice straw	11.500	37.570	432.800	
			11.900	7.520	89.300	
		Bagasse	6.500	7.200	54.400	
		Cassava stem	15.100	2.280	34.500	
		Maize trash	16.600	15.000	248.400	
		Cane trash	15.100	2.400	37.200	
		Peanut shell	18.400	0.180	3.000	
		Firewood	14.800	27.600	407.400	
		Coconut shell	15.800	0.140	2.200	
		Coffee husk	15.500	0.400	6.200	
		Wood residues	7.600	4.080	30.800	
**Cambodia**	2011	Corn cob	16.630	0.090	1.570	[22,23,27,28,29,30]
		Rice straw	14.000	2.070	28.920	
		Rice husk	12.850	1.690	21.730	
		Plywood	-	-	0.043	
		Saw wood	-	-	0.024	
		Logging residues	7.400	-	0.577	
		Cassava stalk	16.990	0.192	3.260	
		Groundnut shell	14.710	0.025	0.373	
			11.230	0.008	0.086	
		Bagasse	6.430	0.035	0.228	
		Coconut front	14.550	0.016	0.229	
		Coconut shell	16.430	0.011	0.184	
**Lao PDR**	2011	Corn cob	16.630	0.110	1.870	[25,31,32,33]
		Rice straw	14.000	0.880	12.310	
		Rice husk	12.850	0.670	8.560	
		Logging residues	7.400	-	0.955	
		Cassava stalk	16.990	0.015	0.260	
		Plywood	-	-	0.256	
		Saw wood	-	-	0.728	
		Bagasse	6.430	0.054	0.349	
**Brunei Darussalam**	2012	Corn fiber	17.250	128.280 × 10^−6^	2.213 × 10^−3^	[22,23,34,35]
		Coconut fiber	19.850	25.101 × 10^−6^	0.498 × 10^−3^	
		Coconut shell	19.400	11.812 × 10^−6^	0.229 × 10^−3^	
		Sawdust	21.500	-	-	
		Rice husk	16.370	351.204 × 10^−6^	5.749 × 10^−3^	
**Indonesia**	2012	Log cutting residues	-	3.705	15.643	[22,24,29,36,37]
		Palm oil fruit empty bunches	-	-	138.300	
		Wood industry	-	7.860	83.840	
		Saw timber	-	4.200	42.000	
		Palm shell	-	-	54.800	
		Bagasse	-	-	129.800	
		Rubber small log	-	-	36.300	
		Coconut shell and fiber	-	-	40.700	
		Corn cob	-	-	71.500	
		Rice husk	-	-	143.300	
**Myanmar**	2012	Lumber waste	-	1.500	-	[22,23,29]
		Molasses	-	0.240	-	
		Bagasse	6.430	2.126	13.670	
		Rice husk	12.850	4.392	56.437	
		Municipal solid waste	-	2.050	-	
**Singapore**	2016	Biomass	-	2.630 (in 2012)	2.901	[22,38]
**Philippines**	2017	Sugarcane waste	-	2.520	-	[22,29,39,40]
		Rice straw	-	5.000	-	
		Rice husk	-	2.000	-	
		Bagasse	-	6.400	-	
		Maize cobs	-	1.000	-	
		Coconut husk	-	6.000	-	
**Thailand**	2018	Rice straw	12.330	10.728	132.300	[22,29]
		Bagasse	7.370	7.645	56.300	
		Sugarcane leaves and tops	15.480	7.811	120.900	
		Rice husk	13.520	4.598	62.200	
		Corn leaves and stems	9.830	3.269	32.100	
		Palm trunk	7.540	1.442	10.900	
		Cassava roots	5.490	4.172	22.900	
		Corn cobs	9.620	0.957	9.200	
		Root, stump and rubber tree branches	6.570	0.808	5.300	
		Palm empty fruit bunch	7.240	2.370	17.200	
		Rubber wood ship and sawdust	6.570	0.485	3.200	
		Palm leaves and branches	1.760	10.529	18.500	

**Table 2:** Resources of agriculture and forest for biomass energy in the SACs.

Year	Rising Area (Hectares/Year × 1000)	Yearly Production (Million Tons/Year)	Percent of Land Area (%)	Forest/Crop	Notes	Refs
-	31,773.000	-	48	Forest	Forest sector can make 1430 Mm^3^ of stock yearly.	[18,19,23,24,28,29,30]
-	13,869.000	-	20.500	Other	-	
-	20,113.000	-	29.730	Other wooded land	Forest sector can make 1430 Mm^3^ of stock yearly.	
-	1903.000	-	2.810	Waterbody	-	
2008	931.000	-	-	Ethanol producible crops	Ministry of Irrigation and Agriculture	
2009	6500.000	-	-	Biodiesel producible crops		
2013	6872.400	19.188 (paddy)	-	Rice	-	[18,28,29,30]
2010	10,094.000	-	57.000	Forest	Forest sector can make 959 Mm^3^ of stock yearly.	
-	40,000.000	0.250	-	Old rubber trees	-	
-	-	0.070	-	Coconut	-	
2014	-	0.550	-	Maize	-	
-	-	2.180	-	Cassava stalk	-	
-	4–10.000	-	-	Oil palm	-	
-	-	0.0240	-	Groundnut	-	
2017	-	10	-	Rice	-	
-	1.000	-	-	Jatropha	-	
-	20.000	0.140	-	Sugarcane	-	
-	13,797.000		44.000	Forest	Forest sector can make 87 Million m^3^ of stock yearly.	[19,28,29,30]
-	7665.000		26.000	Forest	Forest sector can make 1278 Million m^3^ of stock yearly.	
-	1400.000		-	Bamboo	Vietnam makes up to 13 tons of bamboo per ha.	
-	3200.000	-	-	Plantation	-	
-	13,000.000		47.000	Agricultural crops	A land of agriculture crops has 30 million ha.	[18,19,24,28,29,30]
2015	1200.000	16.000	-	Rice	-	
-	-	16.000	-	Biomass residues	Biomass residues comprise the residues from coconut, rice, palm oil, wood, and sugar industries.	
-	380.000	-	-	Sugarcane	-	
-	15,751.000		68.000	Forest	Forest sector can make 929 Mm^3^ of stock yearly.	[19,23,24,28,29,30]
2017	-	1.193	-	Maize	-	
	-	0.218	-	Rice	-	
2012	20.490	1.056 × 10^−3^	-	Sugarcane	-	
	0.440	1.061 × 10^−3^	-	Cassava	-	
-	380.000	-	72.000	Forest	Forest sector can make 72 Mm^3^ of stock yearly.	[19,28,29,30]
-	27,072.000	-	-	Agriculture	-	
-	18,972.000		37.000	Forest	Forest sector can make 783	
-	11,270.000	-	-	Paddy	-	
-	4420.000	-	-	Perennial crop	Recurrent crops include para rubber (3.31 million ha), eucalyptus oil (0.51 million ha), and palm (0.6 million ha).	
-	5020.000	-	-	Field crop	Field crops comprise maize (1.65 million ha), sugarcane (1.67 million ha), and cassava (1.7 million ha).	
-	1540.000	-	-	Orchard	Orchard comprises mixed longan (0.19 million ha), coconut (0.19 million ha), and fruit (1.16 million ha).	
-	94,432.000	-	52.000	Forest	Forest sector can make 11,343 Million m^3^ of stock yearly.	[18,19,23,24,28,29,30]
2011	3808.263	3.267	-	Coconut	-	
	8430.026	19.760	-	Palm oil	-	
	448.745	2.694	-	Sugar	-	
	4131.676	18.328	-	Corn	-	
	12,147.637	66.412	-	Rice	-	
	3445.121	2.592	-	Rubber	-	
-	2.000	-	3.000	Forest	-	[19,23,24,28,29,30]
-	20,456.000		62.00	Forest	Forest sector can make 4,239	[19,28,29,30]
-	4890.000		14.900	Agriculture	-	
2009	-	0.700	-	Sugarcane	-	
	-	0.459	-	Coconut	-	
	-	90.070	-	Palm oil	-	
2015	-	7.772	-	Municipal solid wastes	-	
2017	-	3.5	-	Rice	-	

**Table 4:** Comparative characteristics of thermal power plants using various renewable energy sources.

Characteristics	Unit	Combined Heat and Power by Biomass	Wind Farm (Onshore)	Wind Farm (Offshore)	Photovoltaic Power Plant
Electric power	MW	20	3	5	>1
Investments	Euro/kW installed power	2100–3350	950–1300	1100–3500	1000–2000
Duration of the period of operation	Days a year	182.5–313.9	53.7–98.5	91–175.2	32.8–65.7
Lifetime	Years	20–40	12–30	12–30	15–30
Duration of construction	Years	0.7–3	0.2–1	0.5–2	0.2–1
Production and operating costs	Euro/kW/year (avg.)	150	50	120	33

## Data Availability

Not applicable.
